# Self-Concept in Adolescents—Relationship between Sport Participation, Motor Performance and Personality Traits

**DOI:** 10.3390/sports5020022

**Published:** 2017-04-04

**Authors:** Markus Klein, Michael Fröhlich, Eike Emrich

**Affiliations:** 1 Institute of Sports Science, Saarland University, Saarbruecken 66123, Germany; e.emrich@mx.uni-saarland.de; 2 Department of Sport Science, University of Kaiserslautern, Kaiserslautern 67663, Germany; michael.froehlich@sowi.uni-kl.de

**Keywords:** motor performance, personality traits, self-concept, sport participation

## Abstract

The relationship between sport participation, personality development, self-concept and self-esteem has been discussed repeatedly. In this research, a standardized written survey together with tests on motor performance were carried out with 1399 students (707 male; 692 female) in school years 7 (12.9 ± 0.6 years) and 10 (15.8 ± 0.6 years) to measure the extent of a relationship between physical self-concept (self-developed short scale) and sporting activity, measured motor performance (German motor performance test DMT (Deutscher Motorik-Test) 6–18) and report mark in physical education. Relationships were also analyzed between physical self-concept and general personality traits (neuroticism, extraversion, openness to experiences, compatibility, and conscientiousness, measured with NEO Five Factor Inventory (NEO-FFI)). The assessment of own physical attractiveness and own athleticism differs by sex (*F*(1, 962) = 35.21; *p* < 0.001), whereby girls assess themselves more critically. Weak significant relationships are displayed between motor performance and the assessment of own physical attractiveness (*r*(395) = 0.31; *p* < 0.01). Motor performance is given a higher predictive value with regard to a subject’s own self-concept, (physical attractiveness *β* = 0.37; *t*(249) = 5.24; *p* < 0.001; athleticism *β* = 0.40; *t*(248) = 6.81; *p* < 0.001) than the mark achieved in physical education (physical attractiveness *β* = −0.01; *n.s.*; athleticism *β* = −0.30; *t*(248) = 5.10; *p* < 0.001). Relationships were found overall between personality traits and physical self-concept. The influence of the ‘neuroticism’ trait is particularly strong (physical attractiveness *β* = −0.44; *t*(947) = −13.58; *p* < 0.001; athleticism *β* = −0.27; *t*(948) = −7.84; *p* < 0.001). The more pronounced this trait, the lower the assessment of own physical attractiveness and own athleticism.

## 1. Introduction

The relationship between sport participation, personality development, self-concept and self-esteem has repeatedly been discussed within the framework of legitimation discussions in the sector of school sport. The research shows contradictory findings (see [1,2,3]). These contradictions concern, inter alia, the direction of causal associations of the investigated features. Sport and physical activity are often attributed in general with personality development impact, but this is not clearly and empirically established in this generalized point of view. Character development is often mentioned (e.g., in many physical education curriculum documents, such as [4,5] see also [6]) but no underlying concepts are explained to us that could enable empirical testing. The idea of “educational physical education” [7] is, among other things, based on precisely these attributed effects. Thus, it remains an empirical question to prove or refuse these assumed effects, which cannot be answered in a general and simple point of view. Therefore, the aim of the present paper is to investigate the relationship between sports activity, motor performance and physical self-concepts. The extent to which the physical self-concept is influenced by stable personality traits is another subject of this study. The linking of the self-concept approach with the older, partly controversial trait approach should illuminate some new aspects. One further aim is the construction of a short scale to measure two important aspects of the physical self-concept, which are highlighted in this paper. One is the assessment of own physical attractiveness, the second is the assessment of own motor performance (general athleticism).

## 2. Theoretical Framework

### 2.1. Motor Performance and Self-Concept

The influence of motor activity on the physical development of children is a common place subject in scientific research [8,9,10]. Children ‘grasp’ and experience the world around them using motor perceptive processes, primarily in their early development. Consequently, both the acquisition and the consolidation of new everyday motor and sports motor skills and competences are seen in close connection with the aspects of perception and development. Thus, the expression of different skills and motor actions is directly connected to the development of the nervous system, neuron systems in the brain and the myelination of nerve fibres [11]. We often speak of perceptive-motor development instead of singular motor development because of the close association of motor aspects with those of perception and development. Sports performance motor activity is also implied to have a health-promoting effect within the meaning of bio-psycho-social health and psychological well-being [12].

Psychological well-being also depends on how people perceive and assess themselves in relation to their own appearance and their physical potential. In this respect, we expect relationships between self-concept and psychological well-being. Furthermore, it should be possible to find important indicators for psychological well-being [13,14]. Self-concept comprises knowledge and the assessment of own competence and ability as well as the evaluation of further traits that directly or indirectly affect one’s own self. Shavelson, Hubner, and Stanton [15] see self-concept as a subjective theory about the self that is structured hierarchically and that develops in interaction with the environment and relevant interacting persons (significant others) [15]. At the apex of the hierarchy is a general self-concept which can be divided into two different components, the academic self-concept on the one hand and the non-academic self-concept on the other hand. Physical self-concept is an important part of the non-academic self-concept. This is directed partly towards a person’s own body and appearance and makes reference to the assessment of the perceived effect of one’s own body on others (“physical attractiveness”). In the context of sport and physical performance, the self-assessment of one’s own physical performance (“athleticism”) should have great importance, whereby intensive analysis with one’s own person and individual differentiation of the physical self-concept begins in the puberty phase (between the ages of 10 and 12) [1,16,17].

The general self-concept is formed through external (social) and internal (dimensional) comparisons [18]. Analogously to Marsh [18], schoolchildren use performance feedback from different areas for this purpose, namely a comparative, external reference framework for comparison with performance feedback (e.g., marks) of fellow pupils (social comparison) and an internal reference framework for the evaluation of performance feedback (marks) with regard to own performance (dimensional comparison).

Following Dickhäuser, Seidler, and Kölzer [19], clear positive influences exist with regard to the prediction of academic self-concept in a certain area based on performance in this area (marks in school), which points to the importance of social comparison processes. The dependence of self-concept on the performance level of the comparison group is referred to as the ‘Big-Fish-Little-Pond-Effect’ [20].

Gerlach [21] was able to prove this effect empirically for the physical self-concept, whereby this varies significantly depending on sporting participation. Thus, we can assume that both an evaluation of motor performance (e.g., mark in physical education) and own motor performance must have an explanatory value for the physical self-concept.

With regard to the physical self-concept in relation to sporting activity, adolescents use the motor performance of their fellow pupils as a reference frame in a social comparison and their own performance in this or another sport in a dimensional comparison as a reference frame based on the current time for a comparative evaluation. The inclusion of a positive development that is expected in the future and/or the comparatively weaker development of the performance of others is a possible way to put the results of the social comparison into perspective.

### 2.2. Self-Concept, Sport Participation, Physical Ability and Personality

Self-concept is influenced by personality factors, whereby positive relationships are shown in particular between self-esteem (affective-evaluative components of the self-concept) and extraversion as well as between self-esteem and emotional stability [22]. Moderately positive relationships were found between self-concept (here also specifically the athletic domain of self-evaluation) with extraversion and openness for experiences (intellect). A slightly positive relationship was found with emotional stability [23]. Thus, adolescents with a negative self-concept were more likely to have problems dealing with development challenges than adolescents with a positive self-concept [24]. (Several studies could replicate the big five model in children and adolescents, e.g., [25,26]).

According to Conzelmann and Müller [2], no convincing proof has yet been provided that sport actually promotes personality development. However, the available data suggests that cognitive personality traits (e.g., self-concept) can be influenced more strongly through sporting activity than by static personality traits (global traits) [2,3,27]. Nevertheless, the causal direction here is unclear and cannot be clarified through the methodological approach [28,29].

In another cross-sectional study, it was possible to show differences in the manifestation of the physical self-concept between adolescents who actively practised sport and those who did not [24]. Obviously, sporting activity offers adolescents the opportunity to try out their physical abilities and skills and thus to build up a positive physical self-concept, independent of the setting in which this sporting activity is carried out [24].

Gerlach and Brettschneider [30] were not able to show any interaction between the sporting activity and duration of the sporting activity in a longitudinal study. Those who actively practised sport displayed higher characteristic values at all survey times with regard to their assessment of own sporting activity, which would rather point towards the selection hypothesis and against a change caused by sporting activities or their duration. In contrast, Alfermann et al. [1] found that the assessment of own physical performance sometimes changed in a longitudinal study of adolescents who were active in sports, which would support the hypothesis of change. As for the academic self-concept, various model representations of modes and directions of action are possible in theory. Thus, the Self Enhancement approach indicates that self-concept influences performance while the Skill Development model assumes the reverse, i.e., that the self-concept is formed as a result of the performance [31]. A third idea presumes a two-way causal direction and is called the Reciprocal Effect model (REM, see [17]).

However, Asendorpf and Teubel [32] only found evidence for the Skill Development approach (in general, see [33]) based on the data of the LOGIK study [34,35] in the area of motor skills.

### 2.3. Objective of the Study

More extensive proceedings [36,37] will be used to construct a more economical short scale based on the structure of physical self-concept with the two aspects of ‘general athleticism’ and ‘physical attractiveness’ and to test this short scale by test-statistical analysis, with regard to the gender-specific differences (economical research constraints limited the use of the established methods; thus, the short scale constructed here was part of a comprehensive questionnaire for pupils for which the pupils only had a quite limited time framework).

Subsequently, relationships will be investigated between self-concept and sporting activity on the one hand and motor performance on the other hand. An analysis will be also conducted on the relationships between self-concept and general personality traits, as summarized in the five-factor inventory (neuroticism, extraversion, openness to experience, compatibility, conscientiousness [38,39]).

## 3. Materials and Methods

### 3.1. Subjects

The data is taken from the project ‘Motor performance of children and adolescents in Saarland’, which has been carried out since 2009 in four annual data collection waves [40]. For this presentation, the data material was used from the third wave of data collection in 2011, as this was the first wave in which both a parent questionnaire and a pupil questionnaire were carried out combined with a motor skills test. All students or their parents provided written informed consent to take part in the study that had been approved by the Saarland Ministry of Education (Saarbruecken, Germany). The study complies with the ethical guidelines of Saarland University (Saarbruecken, Germany). Overall, information was provided in the third wave of collection for 1399 pupils (707 male; 692 female) in the 7th (12.9 ± 0.6 years) and the 10th school year (15.8 ± 0.6 years). The case numbers of the individual subsamples and intersections are shown in Table 1.

### 3.2. Variables and Instruments

In order to measure the variables of physical self-concept, a scale was developed modelled after Stiller, Würth, and Alfermann [37] and Gerlach, Trautwein, and Lüdtke [36], partly based on Harter [41], which connects the two dimensions of physical attractiveness and general athleticism. The construction of this short scale was necessary because the written questionnaire was to be completed in the classroom during the lesson (main criterion for this procedure was accessibility of the subjects). The German version [38] of the NEO Five Factor Inventory (NEO-FFI) [39] was used to measure the five personality factors of neuroticism, extraversion, openness for experience, compatibility and conscientiousness. The structure of five factors of personality traits is in several studies replicated for children and adolescents [25,26]. Roth [42] found in all scales of the NOE-FFI good to satisfactory internal consistencies concerning the applicability in children and adolescents. Among other things, the parent questionnaire asks for the mark in physical education in the last half year report before the data collection. The pupils were also questioned about their sporting activities, whereas in the context of the present study the only distinction between “active in sports” versus “not active in sports” was of interest to indicate sport participation.

Motor performance was measured using the German motor performance test DMT (Deutscher Motorik-Test) 6–18 [43]. This test comprises eight test items with which the individual motor dimensions are to be indicated (for objectivity and test-retest reliability see [44]). In detail, these are aerobic endurance (6-min run), strength endurance (push-ups in 40 s and sit-ups in 40 s), explosive strength (standing long jump), action speed (20 m sprint), coordination (under time pressure: jumping side to side; for the precision aspect: balancing backwards) and trunk flexibility (forward bend).

Based on the z-transformed data, an overall motor skill index was formed as an average of the seven items excluding the forward bend (motor index). The test values for the forward bend were excluded from the overall motor index because the mobility is not actually a motor skill in the classical sense (see also [45]).

### 3.3. Statistics

The statistical processing of the data was carried out using IBM SPSS Statistics Version 19 (IBM, Chicago, IL, USA). In order to test the body concept scales, a principal component analysis (PCA) was first carried out with Varimax Rotation to test dimensionality and Cronbach’s Alpha to test the internal consistency of the scale. Part-whole corrected item-total correlations were calculated for each of the items and a one or two factor variance analysis was used for the differentiation tests depending on the number of independent variables. Then, the effect size was quantified using the standardised measurement eta^2^ (*η*^2^). Modelled after the conventions of Cohen [46], effects between 0.01 and 0.059 were classified as small, between 0.06 and 0.139 as moderate and over 0.14 as large effects. Product-moment correlations were calculated in order to test relationships. The relative relationships between different independent variables were determined with multiple linear regressions (forced entry method) with the expression of body-concept as a dependent variable. The relevant prerequisite checks, such as normal distribution, variance homogeneity, sphericity etc., were tested with the appropriate test procedures.

## 4. Results

### 4.1. Scale Analyses

The body concept scale comprises a total of 15 items. The PCA resulted in an interpretable two-factor solution with 60% variance clarification that can be interpreted as a scale of physical attractiveness and a scale of general athleticism (Cronbach’s α each by 0.9). The overall finding was a good part-whole corrected item-total correlation (Table 2). Taking into account the relevant pooling of the items, we can thus form two sum indices (sum scale ‘physical attractiveness’ and sum scale ‘general athleticism’, referred to hereinafter as ‘athleticism’). The totaled score on the ‘physical attractiveness’ scale varies from a minimum of 9 to a maximum of 45. The ‘athleticism’ scale varies from a minimum of 6 to a maximum of 30. The scale averages for ‘physical attractiveness’ were *M* = 35.00 (*SD* = 8.89, *N* = 574) and for ‘athleticism’ they were *M* = 24.31 (*SD* = 6.88, *N* = 577).

### 4.2. Sex and Age

There are gender differences for both scales. The female respondents assessed themselves lower on average than the male respondents in both the trait ‘physical attractiveness’ and the trait ‘athleticism’. Accordingly, girls were on average less satisfied with their bodies and motor performance. As age-specific effects must often be supposed on psychometric scales, the variable age was also tested. The result of the analysis showed neither significant age-specific main effects nor corresponding significant interaction effects (Table 3). Only the main effect gender showed a significant effect.

### 4.3. Sporting Activity

Children and adolescents who are active in sports assess themselves more highly both in the trait ‘physical attractiveness’ and in the trait ‘athleticism’ than those who are not active in sports (physical attractiveness: active in sports: *M* = 32.09, *SD* = 8.43, not active: *M* = 28.31, *SD* = 8.96; *F*(1, 981) = 20.65, *p* < 0.001, *η*^2^ = 0.02; athleticism: active in sports: *M* = 21.88, *SD* = 5.39, not active: *M* = 14.84, *SD* = 4.93; *F*(1, 982) = 181.97, *p* < 0.001, *η*^2^ = 0.16).

As significant effects are seen both in the case of the variable sex and in the variable sport participation, interactions between the two variables were tested using two-factor variance analysis with the two factors sex and sport participation (see Table 4). There are two significant but rather weak main effects in the case of ‘physical attractiveness’ but no significant interaction effect, while for ‘athleticism’ all the effects were significant. While the main effect ‘sporting activity’ displayed a rather large effect size, the effect size of the main effect sex and the interaction effect are very small.

The two previously reported results were also tested with multivariate analysis of variance (MANOVA), which confirm the above (Sex: Pillai’s Trace = 0.02; *F*(2, 947) = 9.87, *p* < 0.001; *η*^2^ = 0.02; Sporting activity: Pillai’s Trace = 0.16; *F*(2, 947) = 88.04, *p* < 0.001; *η*^2^ = 0.02; Interaction Sex x sporting activity: Pillai’s Trace = 0.01; *F*(2, 947) = 3.12, *p* = 0.045; *η*^2^ = 0.01; all other main and interaction effects are not significant).

### 4.4. Motor Performance

The relationships between motor test performance and the two physical self-concept variables showed weak significant correlations for the assessment of physical attractiveness (*r*(395) = 0.31; *p* < 0.001) and moderate effects relating to the assessment of athleticism (*r*(395) = 0.41; *p* < 0.001). It was only for the individual item forward bend that no significant relationship was shown with the values on the scale for physical attractiveness.

Subsequently, the tests for relationships between the two sexes were carried out separately. This showed that the relationship is displayed somewhat more strongly for boys in relation to assessment of physical attractiveness (boys *r*(204) = 0.40; *p* < 0.001, girls (*r*(185) = 0.27; *p* < 0.001). With regard to athleticism, relationships tended to be greater for girls than for boys (boys *r*(205) = 0.53; *p* < 0.001, girls (*r*(185) = 0.58; *p* < 0.001), whereby the extent of the correlations did not differ significantly between the sexes in either case.

A multiple linear regression was calculated in order to test the level of influence of the motor performance and the mark in physical education. The results show that motor test performance has a substantially greater predictive value with regard to the self-assessment of athleticism for both sexes than the mark in physical education (Table 5). The variance clarification was much weaker for the scale of physical attractiveness. Although the motor test performance had a significant, if weak, influence on the perception of own physical attractiveness for both sexes, the mark in physical education did not play a role for either.

### 4.5. Personality Traits

Multiple regressions were calculated in steps for both sexes to test the relationship between individual personality traits with the assessment of physical attractiveness and athleticism (Table 6). The most noticeable thing here is the high negative importance of neuroticism. This shows the strongest relationship with the assessment of physical attractiveness and athleticism. A less strong relationship is shown in the case of extraversion with the assessment of athleticism. If we look at the relationships in a gender-specific process then there is a noticeable, slight gender-specific difference in the relationship with the scale of athleticism. For girls, the level of relationship between extraversion and athleticism is somewhat higher than that between neuroticism and athleticism, while this is reversed for boys.

## 5. Discussion

The short scale for the measuring of the physical self-concept comprises a total of 15 items. The assessment of own physical attractiveness was measured using nine items, the assessment of own athleticism using six items. The item analysis resulted in a good scale property, so that the use of the measurement instrument for further analysis appears to be justified.

The assessments both of own physical attractiveness and own athleticism differ by gender. Girls see themselves somewhat more critically and/or they are somewhat less satisfied than boys [1]. The findings also fit with possible gender-specific differences in motive for taking up sporting activities. Thus, we can certainly see higher participation in organised competitive sports for boys and somewhat higher participation in self-organised and non-competitive sport for the girls [47]. However, the fact that no effects were shown with respect to age goes against the currently available findings [1] and thus against the assumption of a clear differentiation of physical self-concepts during the puberty phase.

With regard to sport participation, there was a difference in both the assessment of own physical attractiveness and the assessment of own athleticism. Both were assessed somewhat more positively by adolescents who are active in sports than by those who are not active. It is, however, not possible to establish a causal relationship in the form of an effect of sports participation on the physical self-concept from the data. Competing explanations are, on the one hand, an influence on the physical self-concept through sporting activity (socialisation hypothesis) and, on the other hand, taking up sporting activity or sports participation because of a better physical self-concept (selection hypothesis). Burrmann [24] was able to supply findings that were more in favour of the socialisation hypothesis. Overall, it was not possible to determine an important interaction between sport participation and sex.

The two recorded aspects of the self-concept also correlate with objectively measured motor test performance. Weak, but still statistically significant relationships are displayed between motor skill performance and the assessment of own physical attractiveness. As we have already argued that findings in the literature favour the socialisation hypothesis (self-concept changes through sports participation [24]), the relationships between the objective motor performance level and measured self-concept aspects can be associated with the Skill Development model. This was also the conclusion of Asendorpf and Teubel [32], who were able to prove this using longitudinal data from the LOGIK study [34,35]. It was also shown in this context that motor testing with regard to self-concept had a higher predictive value for self-concept in the area of athleticism than the mark in physical education. This situation leads us to assume that children and adolescents assess their self-concept quite realistically and relate the assessment of their athleticism primarily to individual performance. The report of school performance seems to play a subordinate role in the formation of the physical self-concept. The rather insignificant influence of the mark in physical education in this context also corresponds to findings within the framework of the discussion regarding the diagnostic competence of teachers [48]. If we pay attention to the specific aspects of self-concept that are studied in this context, then we see a tendency for boys to define their physical attractiveness more strongly based on their athleticism than girls. On the contrary, girls seem not to use the reference framework of school sports when assessing their physical attractiveness but rather to refer to other social contexts. Analogously to Rost and Sparfeldt [49], we cannot, however, speak of general gender stereotypes here. The results rather point towards the necessity of a differential observation of sex in the context of the self-concept.

Overall, we were able to show that personality traits determine the physical self-concept to a certain extent. This aligns with the findings on the general self-concept [22]. The influence of the trait of neuroticism is particularly highly substantial. The more strongly this trait is expressed (so the lower the emotional stability), the lower the assessment of own physical attractiveness and own athleticism. Reference group effects are of particular interest in further evaluation steps. Thus, it has already been possible to show that the sporting and/or physical self-concept of individuals in a high-performance environment (e.g., a school class with particularly athletic children) tends to be low [21,34]. This corresponds to the ‘Big-Fish-Little-Pond-Effect’ (BFLPE) that was already discussed and proved in the 1980s with regard to the academic self-concept [20]. Further research will examine supplementary influence variables e.g., parenting style and parenting goals.

## Figures and Tables

**Table 1:** Subsamples and Intersections (Partial evaluation of the 2011 data collection wave).

**All**	**Boys**	**Girls**	**Total**
	707	692	1399
Parent questionnaire	390	380	770
Pupil questionnaire	498	486	984
Motor performance test	387	340	727
**Intersections**			
Parents & Pupils	213	206	417
Pupils & Motor skills	240	220	457
Parents & Motor skills	273	232	506
Parents & Pupils & Motor skills	158	144	299

**Table 2:** Scale values and factor loading, (+) means the item remains in its original poling, (−) means the polarity of the item must be reversed (α = Cronbachs α; *r*_it_ = part-whole corrected item-total correlation, bold values indicate factor loading > 0.50).

Item	*M*	*SD*	Factor 1	Factor 2	*r*_it_	Scale Value
I like my body. (+)	3.58	1.21	**0.84**	0.23	0.81	*α* = 0.90 (*n* = 928)
I am satisfied with my appearance. (+)	3.65	1.17	**0.78**	0.21	0.73	
I like to show my body. (+)	2.68	1.21	**0.55**	0.25	0.51	
There are many situations in which I am satisfied with my body. (+)	3.79	1.18	**0.63**	0.21	0.58	
I consciously choose my clothing to hide my body. (−)	3.74	1.29	**0.57**	0.06	0.48	
I often feel uncomfortable in my body. (−)	3.82	1.25	**0.77**	0.11	0.70	
I would like to have a different body. (−)	3.66	1.37	**0.84**	0.14	0.79	
If I could change something about my body I would. (−)	3.06	1.48	**0.76**	0.06	0.66	
I think I am too fat. (−)	3.69	1.44	**0.77**	0.19	0.73	
I am very good at sport. (+)	3.45	1.28	0.19	**0.86**	0.81	*α* = 0.89 (*n* = 928)
I learn faster in sport than other people my age. (+)	3.00	1.20	0.14	**0.79**	0.70	
I learn new sport exercises very quickly. (+)	3.74	1.13	0.12	**0.78**	0.69	
I am just not good at sport. (−)	4.02	1.18	0.22	**0.73**	0.66	
I find most sports easy. (+)	3.61	1.13	0.14	**0.80**	0.71	
Others are better than me at sport. (−)	3.24	1.29	0.18	**0.75**	0.68	

**Table 3:** Scale values ‘physical attractiveness’ and ‘athleticism’ and results of the two-factor analysis with the factors class (as an indicator for age) and sex.

**Scale**	**Sex**	**Class 7**			**Class 10**		
		***n***	***M***	***SD***	***n***	***M***	***SD***
Physical attractiveness	Male	314	32.66	8.63	175	34.06	7.00
	Female	295	29.59	9.32	180	30.46	7.67
Athleticism	Male	313	22.18	5.48	177	22.92	5.86
	Female	295	19.79	5.40	180	18.89	5.90
**Two-factor ANOVA**							
**Scale**		**Factors**		***df***	***F***	***p***	***η_p_*^2^**
Physical attractiveness		Sex		1	35.21	<0.001	0.04
		Class		1	4.09	0.043	0.00
		Sex x class		1	0.23	0.645	0.00
*n* = 964; *R*^2^ = 0.04							
Athleticism		Sex		1	73.60	<0.001	0.07
		Class		1	0.05	0.830	0.00
		Sex x class		1	4.75	0.030	0.00
*n* = 965; *R*^2^ = 0.07							

**Table 4:** Result of the two-factor variance analysis with the factors sex and sporting activity.

Scale	Factors	*df*	*F*	*p*	*η_p_*^2^
Physical attractiveness	Sex	1	12.56	<0.001	0.01
	Sporting activity	1	15.20	<0.001	0.02
	Sex x sporting activity	1	0.08	0.782	0.00
*n* = 967; *R*^2^ = 0.06					
Athleticism	Sex	1	12.94	<0.001	0.01
	Sporting activity	1	182.97	<0.001	0.16
	Sex x sporting activity	1	5.59	0.018	0.00
*n* = 968; *R*^2^ = 0.22					

**Table 5:** Summary of the multiple regressions for the prediction of the scale values ‘athleticism’ and ‘physical attractiveness’ (influence of the motor performance and the mark in physical education).

**Variable**	**Scale ‘Physical Attractiveness’**					
	**Boys**			**Girls**		
	***B***	***SE***	***β***	***B***	***SE***	***β***
(Constants)	−27.26	13.74	-	−7.87	17.09	-
Motor skill index	0.58	0.12	0.45 ***	0.38	0.15	0.27 *
Mark in physical education	0.74	1.14	0.06	−0.42	1.40	−0.03
	*R*^2^ = 0.18 ***			*R*^2^ = 0.09 ***		
**Variable**	**Scale ‘Athleticism’**					
	**Boys**			**Girls**		
	***B***	***SE***	***β***	***B***	***SE***	***β***
(Constants)	−7.80	7.25	-	−15.75	8.75	-
Motor skill index	0.33	0.06	0.43 ***	0.40	0.08	0.44 ***
Mark in physical education	−1.98	0.60	−0.27 ***	−2.43	0.71	−0.29 **
	*R*^2^ = 0.38 ***			*R*^2^ = 0.42 ***		

* p < 0.05; ** *p* < 0.01; *** *p* < 0.001.

**Table 6:** Summary of the multiple regressions for the prediction of the scale values ‘athleticism’ and ‘physical attractiveness’ (influence of the motor performance and the mark in physical education).

**Variable**	**Scale ‘Physical Attractiveness’**					
	**Boys**			**Girls**		
	***B***	***SE***	***β***	***B***	***SE***	***β***
(Constants)	35.457	3.422	-	31.732	4.052	-
Neuroticism	−0.451	0.053	−0.401 ***	−0.475	0.057	−0.403 ***
Extraversion	0.115	0.062	0.086	0.045	0.071	0.030
Compatibility	0.050	0.067	0.033	0.147	0.074	0.090 *
Conscientiousness	0.069	0.050	0.063	0.042	0.055	0.034
Openness	−0.017	0.064	−0.011	0.066	0.064	0.044
	*R*^2^ = 0.23 ***			*R*^2^ = 0.21 ***		
**Variable**	**Scale ‘Athleticism’**					
	**Boys**			**Girls**		
	***B***	***SE***	***β***	***B***	***SE***	***β***
(Constants)	22.872	2.516	-	12.816	2.660	-
Neuroticism	−0.190	0.039	−0.241 ***	−0.117	0.037	−0.155 **
Extraversion	0.185	0.046	0.198 ***	0.277	0.046	0.290 ***
Compatibility	−0.053	0.049	−0.049	−0.080	0.048	−0.076
Conscientiousness	0.044	0.037	0.057	0.101	0.036	0.129 **
Openness	−0.090	0.047	−0.083	0.000	0.042	0.000
	*R*^2^ = 0.16***			*R*^2^ = 0.18***		

* *p* < 0.05; ** *p* < 0.01; *** *p* < 0.001.
